# Behavioral Trajectories During Middle Childhood: Differential Effects of the School-Wide Positive Behavior Support Model

**DOI:** 10.1007/s11121-018-0938-x

**Published:** 2018-08-18

**Authors:** Mari-Anne Sørlie, Thormod Idsoe, Terje Ogden, Asgeir Røyrhus Olseth, Torbjørn Torsheim

**Affiliations:** 1 The Norwegian Center for Child Behavioral Development (NCCBD), P.O. Box 7053, 0306 Majorstuen, Oslo Norway; 2 University of Bergen, Bergen, Norway

**Keywords:** Antisocial, GMM, Longitudinal, Trajectory, SWPBS

## Abstract

The aims of this study were to assess the longitudinal trajectories of externalizing problem behavior during middle childhood among typically developing children and to examine subgroup differences in the effectiveness of the School-Wide Positive Behavior Support (SWPBS) model, called N-PALS in Norway. Participants were approximately 3000 students, and behavioral assessments were performed by class head teachers at four time points from the 4th or 5th grade through the 7th grade. Using a combination of latent class growth analyses (LCGA) and growth mixture modeling (GMM), four distinct trajectory classes were identified, i.e., persistent low (84.4%), persistent high (2.5%), decreasing (7.9%), and increasing (5.3%). An indication of a significant positive effect of the N-PALS model was found for students with a persistently high-risk trajectory. The current study adds to the evidence that this school-wide prevention model can moderate the development of externalizing behavior problems among children and youth.

**Electronic supplementary material:**

The online version of this article (10.1007/s11121-018-0938-x) contains supplementary material, which is available to authorized users.

Externalizing problem behaviors, such as opposition, aggression, harassment, and delinquency, are among the most common, serious, and persistent forms of maladjustment among children and youth (e.g., Ogden and Hagen 2014), although such behaviors change both in frequency and intensity over the course of development (Tremblay 2000). For example, physical aggression normally decreases with age while the opposite is true for truancy and drug and alcohol use (Tremblay 2000). High stability of externalizing behavior problems over time is reported in longitudinal studies covering a wide range of cultural contexts, samples, and time periods (Piquero et al. 2012). However, substantial heterogeneity in the behavioral development is also reported, indicating that there are likely subgroups with different trajectories of externalizing behavior (e.g., Jennings and Reingle 2012; Odgers et al. 2008). Accordingly, knowledge of the average developmental trajectory and single-point assessments are insufficient (e.g., Reef et al. 2011). The challenge of externalizing behaviors has been an issue of concern for school personnel and the topic of numerous research publications (e.g., Bongers et al. 2008; Ogden 2015). Even if research on school-based interventions has documented positive main outcomes, subgroup effects have less often been examined (Split et al. 2013).

The prevalence of serious externalizing behavior is low in most schools, and therefore, much of a school’s preventive effort may be directed at children who are unlikely to be at elevated risk of conduct problems (Humphrey et al. 2013). A combination of universal and individually tailored school interventions are recommended for students showing high levels of externalizing behavior (i.e., high risk of conduct disorder), preferably as part of a whole-school and multi-component approach (Weare and Nind 2011).

It follows that subgroup effects should be examined in multi-tiered school-wide interventions like the School-Wide-Positive Behavior Support (SWPBS) model (Bradshaw et al. 2015). Most evaluations of the SWPBS model have, however, focused on main effects of the universal tier (tier 1) only, while few have, as in the present study, investigated the differential effects of the *combined* three-tiered model (Sørlie and Ogden 2015). Data from a large-scale longitudinal evaluation of SWPBS as implemented in Norway (Sørlie and Ogden 2014) were used to examine the heterogeneity of externalizing trajectories from grade 4 through 7 among students in 48 ordinary Norwegian elementary schools, and to explore possible differential intervention effects on externalizing trajectories. Previously, significant main effects of the full N-PALS model have been reported on several outcomes (Sørlie and Ogden 2015; Sørlie et al. 2016).

## School-Wide Positive Behavior Support in Norway

The main objectives of the N-PALS model are to prevent and reduce externalizing student behavior; establish a positive, supportive, and inclusive school climate; and “foster durable changes in the behavior of higher-needs students” (Bradshaw et al. 2008, p. 3). The N-PALS model is organized as a three-tiered school-wide framework with empirically supported interventions implemented at the universal level (tier 1), the selected level (tier 2), and the indicated level (tier 3). Proactive and positive behavior supports and preventive interventions for the majority of well-behaved students (tier 1) are combined with comprehensive and intensive strategies targeting students showing moderate-to-high levels of problem behaviors (tiers 2 and 3). By matching interventions to the students’ risk level, the prevalence of disruption and acting out behavior is expected to decline, both inside and outside the classrooms.

The three-tier model implemented in the current project had a team-structured organization of school leadership and development activities. A team of five to six representatives was appointed at each of the schools and trained to plan, inform, conduct, monitor, and report on the interventions and outcomes at their school. The school teams were trained and supervised locally by a certified coach for a period of 2 years (2 h for each of 10 training sessions per year). Next, the staff at each school was informed and trained in the key model and implementation features by the school teams.

At the *universal level*, the core components included (1) school-wide positive behavior support strategies in which 3–5 positively formulated school rules were taught and followed up by systematic praise (including reward cards) and supervision from staff (moreover, all students received training in expected prosocial skills), (2) a web-based system for tracking referrals based on systems for monitoring student behavior across all school areas (School-Wide-Information System, SWIS), (3) collectively applied school-wide corrections with mild and immediate consequences (response cost), (4) classroom management skills training for teachers, and (5) parent information and collaboration strategies.

At the *selected* level (tier 2), students who did not benefit sufficiently from the universal interventions were identified and received time-limited small group instruction and training in social skills or the behavioral education program Check-In, Check-Out (CICO) (Todd et al. 2008). Moreover, increased contact and communication between parents and the school was established (e.g., by daily or weekly report cards). At the *indicated* level (tier 3), high-risk students were provided with an individualized support plan based on functional behavior assessment (FBA), which could include individual special education and intensive individual social skills training, e.g., the Stop-Now-and-Plan (SNAP) program (Augimeri et al. 2007). The N-PALS schools used web-based assessment and feedback to ensure that decisions of action were supported by relevant data, and interventions were implemented with fidelity.

## Differential Effects of SWPBS

To our knowledge, only one prior study has investigated differential effects of the SWPBS model on students at varying levels of risk and was, therefore, of particular relevance to the present study. Bradshaw et al. (2015) explored the extent to which the effects of SWPBS varied as a function of the students’ baseline pattern of social-emotional and behavioral risk. In this group-randomized wait-list control study, data were collected across five time points over four school years from 12,334 children in 37 public elementary schools. Using latent profile analyses (LPA), four subgroups (latent trajectory classes) were identified based on the teachers’ baseline ratings of student problem behavior, concentration problems, social-emotional functioning, and prosocial behavior. The high-risk class (6.6%) showed high levels of concentration problems and disruption, with low levels of emotion regulation and prosocial skills. The at-risk class (23.3%) was slightly above the sample mean on concentration problems and disruption and slightly below the mean on prosocial skills and emotion regulation. The normative class (36.5%) generally had scores close to the mean. The socially-emotionally skilled (33.6%) had scores below the mean on concentration problems and disruption, with scores above the sample mean on prosocial and emotion regulation skills. The at-risk and high-risk classes had significantly greater intervention benefits (fewer office disciplinary referrals, less likelihood of receiving counseling for inappropriate behavior, and being referred to special education) relative to their counterparts in the control schools who desisted from implementing SWPBS for 4 years.

The outcomes for students at risk in Bradshaw et al.’s (2015) study match well with results from similar studies in which subgroups were established based on pre-intervention scores. In their review of universal school-based violence prevention programs, Farell et al. (2013) identified 68 studies examining subgroup differences.

Of these, 20 examined how intervention effects varied as a function of the level of aggression at pretest. They summarized that, regardless of the statistical method used, 12 of the 20 studies found greater benefits for individuals at high levels of initial aggression compared to those at low levels.

## Behavioral Trajectories

There is growing evidence of heterogeneity in behavioral trajectories across time, indicating stable heterogeneous externalizing subpopulations (e.g., Jennings and Reingle 2012; Piquero et al. 2012). Moffitt’s (1993) theoretical framework has been influential in the development of knowledge and research on aggressive and antisocial trajectories. She suggested an adolescent-limited trajectory (i.e., onset in adolescence with desistance in adulthood) and a life-course persistent trajectory (i.e., onset in childhood with little desistance over time). This framework has been extended to a variety of externalizing behaviors such as bullying, hyperactivity, violence, physical aggression, opposition, and conduct disorder (e.g., Broidy et al. 2003; Odgers et al. 2008). Others have reported on additional trajectories such as a moderate stable, moderate decreasing, and increasing (e.g., Husemann et al. 2009; Brame et al. 2001). Principles from a theory underlying the SWPBS model (Sørlie and Ogden 2015) may contribute to explain how various trajectories come into being. For example, in line with applied behavior analysis, SWPBS systematically teaches and reinforces appropriate (expected) student behavior in all school areas; changes the antecedents and consequences of appropriate and problem behavior; and, in some cases, adjusts or establish motivating operations. Student behavior influenced by peers or other out-of-school influences may, however, lead to problem behavior within school even after SWPBS implementation, such as a peer serving as a trigger for aggression. Students with challenges in the home may arrive late, tired, and stressed (key motivating operations), which increase the likelihood that they respond with verbal or physical aggression (problem behavior) if rebuked by a teacher (antecedent) to escape the unpleasant situation (consequence). Such out-of-school contributors to problem behavior may lead to the appearance of a persistently high trajectory group in teacher reports. In contrast, an initially high but decreasing trajectory group may have shown externalizing behavior maintained primarily by antecedents and consequences within the school context.

Modern group-based trajectory modeling has identified anywhere from two to seven trajectories of externalizing behavior depending on age or subtypes of problem behavior (aggression, violence, delinquency) (Walters and Ruscio 2013). The relative variability observed in number, size, and shape of the behavioral trajectories across studies may be due to differences in analytic approach, measures, time span, or sample composition (e.g., Jennings and Reingle 2012; Harachi et al. 2006). Such design elements explain why the validity and reliability of some of the prior study findings can be questioned (Shadish et al. 2002). Most studies have identified three or four distinct trajectories that are theoretically justified, distinguishable, and interpretable (e.g., Jennings and Reingle 2012; Odgers et al. 2008; Piquero et al. 2012; Reef et al. 2011).

In their overview, Eisner and Malti (2015) conclude that four important patterns are evident in a majority of the studies. First, most of them found a subgroup of children, mostly boys, demonstrating high and stable aggressive behavior over long developmental time spans. Second, most studies operate with a persistently low group with a sustained low level of aggression over time, and with an overrepresentation of girls. Next, some children are grouped into one or two declining trajectories across early and middle childhood. Finally, there is a group of children with an escalating trajectory between ages 11 and 17 (Jennings and Reingle 2012; Odgers et al. 2008). According to Eisner and Malti (2015), this distinction among four major trajectory groups may be considered an analytically useful and empirically supported classification. It corresponds with Moffitt’s (1993) theoretical classification of longitudinal development from childhood to adulthood, but may also be relevant for the developmental period of middle childhood, as covered by the current study. About 30 to 50% of various samples are found to have a decreasing trajectory of aggression from age nine to 13 while 1.5 to 4% is reported to follow a stable high trajectory (e.g., Brame et al. 2001). Such developmental profiles, supported by theory and empirical studies, may also make sense for practitioners in elementary school.

In the current study, repeated measures were used to allow for inspection of individual trajectories across multiple time points. Such analyses are central in determining for whom the intervention works and to which extent the effects of a specific intervention may be generalized (Flay et al. 2005). Muthén et al. (2002) recommend such a design for investigating the effects of interventions for externalizing problem behavior within a group-based trajectory model. In the current study, differential intervention effects were investigated based on subgroup trajectories estimated in the control group. Compared to Bradshaw et al. (2015) study, which used one assessment point (baseline) only to define trajectory classes, this approach used data from several assessment points and growth mixture modeling (GMM) to be able to discriminate between potential latent subgroups with different slopes but similar baseline levels of externalizing behavior.

Building on theory and as demonstrated in previous studies, the current study was based on the assumption that four distinctive developmental trajectories could be identified, i.e., (1) a stable low trajectory for the majority of students; (2) a high trajectory indicating persistent externalizing problem behavior for a small group of students; (3) a high or medium but decreasing trajectory; and (4) a small group which starts low, but with an increasing trajectory. A latent class and growth approach was used to identify the number of trajectories and potential intervention effects (see Fig. A1 in Appendix, available online). Those on a low-level trajectory were expected to change the least following the implementation of the N-PALS model because of little room for change in this group. Students in the intervention schools with persistently high and escalating trajectories were hypothesized to change the most and to show less externalizing behavior over time than their counterparts in the comparison schools.

## Method

**Fig. 1:**
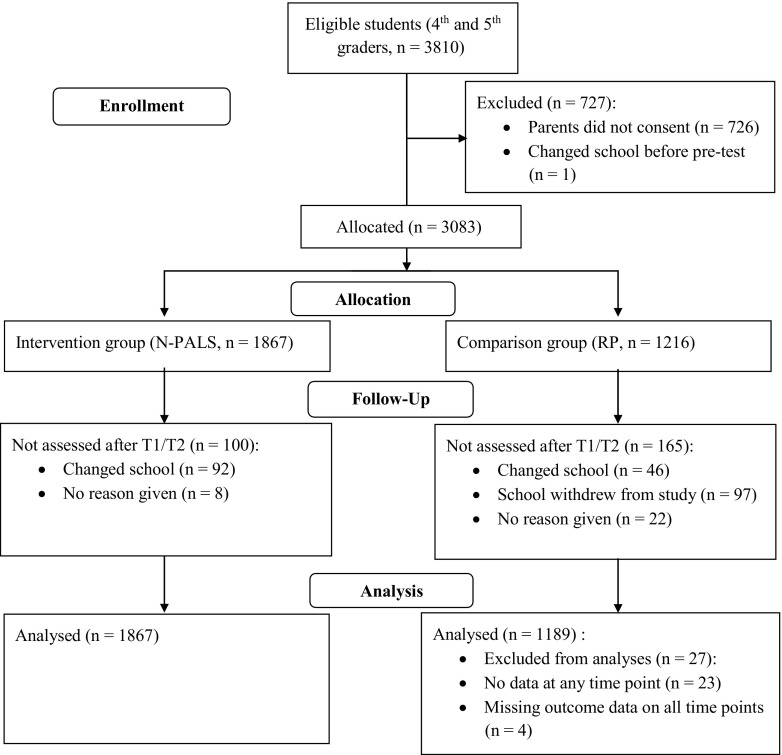
Flow chart adapted from Schulz et al. (2010). Lost to follow-up includes students with a monotone pattern of missing. They were rated by their class head teachers at T1 or T1 and T2 (i.e., at the beginning and/or end of the first of four school years) and then missing on consecutive time points. For the intervention group, all allocated students had outcome data on at least one time point throughout the study period. All students where thus included in the analyses due to the use of full information robust maximum likelihood estimation. For the comparison group, 27 students had missing data either on all variables or on all dependent variables on all time points. Accordingly, these students were excluded from the analyses

Questionnaire data were collected from class head teachers in the 4th to 7th grades at four time points (T1–T4) in 48 Norwegian elementary schools participating in a non-randomized experimental effectiveness study of the N-PALS model (Sørlie and Ogden 2014). Several elements were added to the design to reduce potential threats to the internal validity, and schools were randomly invited to participate as intervention or control schools. Schools that volunteered were selected according to predefined inclusion and exclusion criteria (Sørlie and Ogden 2014). The behavioral development of 4th and 5th graders (9–10 years) in the 28 N-PALS intervention schools were compared through the 7th grade (12 years) with students of the same age in 20 schools that did not receive the intervention (regular practice, see Fig. 1). Schools already implementing interventions to prevent student problem behavior were not included. Baseline (T1) was close to the initiation of the intervention and the beginning of a new school year (August–September). Follow-up assessments were conducted in June after 1, 2, and 3 years of implementation (T2–T4).

### Participants

In order to track a stable group of students over the four assessment points, the present study included all students with parental consent who entered 4th or 5th grade (25%) at the start of the intervention (*N* = 3083) with 61.1% of the student sample in the intervention schools. Fifty-one percent were boys and about 6% had an ethnic minority background. The total staff amounted to 1064 in the intervention schools and 750 in the comparison schools, of which 80% were females and older than 35 years, and 64% were employed as teachers while 4% had no formal training. The mean school size was 282 students, and the student-teacher ratio per class was 1.7. Significant group differences at baseline were found on only one of 29 variables explored (lower mean reading score in the N-PALS group), and the participating schools were fairly representative of Norwegian elementary schools (Sørlie and Ogden 2014).

Data were collected on the internet or paper during the school day. To standardize the assessment procedures, written instructions were given. Informed and written assent from the studentsʼ parents was obtained in advance. Assent from the staff was obtained coincident with completion of the questionnaire.

### Measures

#### Externalizing Behavior

Externalizing problem behavior was measured by the broadband scale (30 items) of the Teacher Report Form (TRF, Achenbach 1991) which taps the subdimensions aggression and delinquency. TRF has demonstrated acceptable reliability and validity in previous Norwegian studies (e.g., Ogden and Hagen 2006). The teachers rated each student in their class on items like “disobedient at school,” “lacks guilt,” “bad companions,” and “alcohol, drugs,” by using a 3-point scale; *not true* (0), *somewhat or sometimes true* (1), or *very true or often true* (2). Higher scores indicate higher levels of maladaptive behavior (max. = 60, min. = 0). In the present study, the reliability ranged from α = .76 to .96 across assessment points.

#### Implementation Quality

Implementation quality was assessed by “The Effective Behavior Support Self-Assessment Survey” (EBS-SAS, Sugai et al. 2009), which has been found sufficiently reliable and valid for the purpose of measuring perceived SWPBS fidelity (e.g., Solomon et al. 2015). The rating scale includes four subsections, i.e., the schoolwide system, (18 items), the non-classroom system (9 items), classroom system (11 items), and individual student system (8 items). Only the sum scale (46 items) was used in the analyses. Items like “A few (3–5) school rules are positively and clearly defined” (school-wide), “Problem behavior is consistently addressed with mild and predictable negative consequences (1:5)” (classroom), “The staff members who have inspection actively supervise the students in all arenas outside the classroom context” (common areas), and “A behavioral support team is immediately established (within 2 days) for students showing severe behavior problems” (individual) were assessed using a 3-point scale (1 = *in place*, 2 = *partly in place*, 3 = *not in place*). All school personnel in the N-PALS schools completed the measure (*α* = .90 to .92.) The most complete staff ratings (T4) were entered in the analyses.

### Analytic Strategy

Following the recommendation of Muthén et al. (2002), growth mixture modeling (GMM) was used to assess intervention effects in this longitudinal trial. GMM is intended to explore potential subgroups where an intervention has effects within studies that have already demonstrated an overall treatment effect (Muthén et al. 2002). The approach identifies latent subgroups through class enumeration procedures and subsequently allows modeling of potential intervention effects for the different subgroups by comparing their trajectory slopes within comparison and intervention samples. Other group-based procedures exist, but GMM allows for within-class variance in the growth factors (intercepts and slopes). Although the average trajectories vary across subgroups, individual variation around each subgroup trajectory is possible (Muthén et al. 2002). GMM makes use of information from all time points to suggest potential subgroups. The inclusion of all time points was preferred in order to discriminate between potential subgroups with similar pretest levels but with different slopes. We followed a stepwise procedure recommended by Muthén et al. (2002). This starts with the assumptions that intervention effects are captured in the average slopes of each subgroup and that a person has a stable trajectory class membership over time. An intervention effect will occur as a change in within-class trajectory that is different from the one expected for the comparison group. To establish normative growth in the absence of the intervention, class enumeration procedures should first be conducted for the comparison group. If the intervention does not influence subgroup membership, the number of latent classes identified in the comparison group should be replicated in the intervention group. After a separate investigation of the number of classes in both groups, a joint analysis should be conducted. Finally, differences between the average slopes for latent classes in the two groups should be tested. If so, this would indicate that the development was significantly different in the intervention subgroup relative to the comparison subgroup.

Using Muthén et al.’s (2002) procedure as the overall framework, we integrated this with recommendations from Jung and Wickrama (2008) that suggest latent class growth analysis (LCGA) as a starting point for conducting class enumeration procedures within GMM. They regard LCGA as a specific type of GMM in that the variance and covariance of the growth factors within each class are constrained to zero. Jung and Wickrama (2008) argue that this is beneficial for the initial class enumeration procedures since it identifies distinct classes prior to GMM and allows for faster model convergence due to the constraints. Whether or not the inclusion of covariates improves class enumeration, procedures are found to vary with sample size and class separation (Hu et al. 2017). Furthermore, if the covariates have significant direct effects on the growth factors (as assumed), the class enumeration process may be distorted. Based on the recommendations of Jung and Wickrama (2008), each of the steps above (LCGA and GMM within comparison, intervention, and pooled samples, respectively) were first conducted without covariates (unconditional models) and then with covariates (conditional models). Although prior research indicates that latent trajectory classes are gender invariant (e.g., Piquero et al. 2012), ignoring levels for gender in the analysis could lead to biased results (Baker and Kramer 2001). The gender issue could be dealt with in either of two ways, by running separate analyses for each gender or by treating gender as a covariate. We chose the latter because splitting the sample on gender would cause too small groups to obtain reliable results. As in previous studies, some instability in the studentsʼ externalizing behavior across middle childhood was also expected (e.g., Tremblay 2000). Accordingly, student gender and age were entered as covariates.

The analytical procedure can be summarized in the following steps: (1) comparison group analyses—class enumeration procedures by running unconditional and conditional models by use of LCGA. Then, a repetition of the same procedures using GMM (allowing within-class variances for the growth factors), (2) intervention group analyses—repeating the procedures from step one to see if the results could be confirmed with the intervention group; (3) joint analyses of the comparison and the intervention group—repeating all the procedures from step one and two to see whether the class enumeration procedures hold for the pooled sample. Treatment condition was also entered as a covariate in the conditional models to investigate whether this affected class membership, (4) after confirming the class enumeration procedure through the three steps above, possible intervention effects were examined in the pooled sample by testing whether treatment condition had significant effects on the trajectory slopes within each subgroup. In case of a dichotomous predictor (as for our treatment conditions), Muthén and Muthén (2014) recommend that effects should be standardized according to the outcome variables only. This can be interpreted as the change in *y* (externalizing behavior) standard deviation units when *x* (treatment condition) changes from zero to one. This is thereby a standardized mean difference in change score across treatment conditions.

Models were fitted to the data with Mplus 7.3 (Muthén and Muthén 2014) using robust maximum likelihood estimation with EM algorithms to account for non-normal variable distributions. In determining the number of latent trajectories, a recommended combination of fit indices, research question, parsimony, theoretical justification, and interpretability was applied (Jung and Wickrama 2008). Entropy values were also investigated. Values closer to 1 indicate good classification quality, i.e., the latent classes are clearly distinguishable. The sum of the indices should be combined with the interpretability and theoretical justifications of a given class solution when deciding on the final model. As the observations were nested within teachers (raters), design-based standard errors were obtained using the Mplus complex option.

## Results

### Descriptive Statistics

Mean values on the TRF externalizing scale in the control group ranged from 4.2 to 4.7 across time points with standard deviations in the range of 8.2 to 8.7 (skewness 3.0 to 3.3, kurtosis 10.4 to 12.5). In the comparison group, the means ranged from 4.4 to 4.9 with standard deviations between 8.2 and 8.7 (skewness 2.8 to 2.9, kurtosis 8.7 to 10.1). For girls, the means ranged from 2.3 to 2.6 with standard deviations from 5.0 to 5.9 (skewness 3.6 to 4.4, kurtosis 16.9 to 25.3). For boys, the means ranged from 6.3 to 7.1 with standard deviations from 9.7 to 10.8 (skewness 2.1 to 2.4, kurtosis 4.9 to 6.0).

### Attrition

Of the 3056 students included in the analyses, the majority (71.4%) were assessed at all time points, 17% on three occasions; 6.5% on two occasions; and 5.1% on one occasion. Missing values ranged from 0 to 14.3%. Patterns in missing over time were examined to detect indications of selective attrition (Enders 2010). The most common was an intermittent pattern where responses were missing on varying time points throughout the study period (*n* = 461). The second most common was a monotone pattern where the response was present on the first and/or second time point but subsequently missing; most (*n* = 201 of 265) because their families moved to other parts of the country. A third pattern consisted of those missing on the first time point but present on all following time points (*n* = 148). Nominal logistic regression models with treatment group, gender, grade, baseline externalizing problems, and interaction between baseline externalizing problems, and treatment group as predictors indicated that individuals in the control group had a higher likelihood of being in any of the three missing pattern groups. Girls were more likely to be in the intermittent group, and higher levels of externalizing were related to a greater likelihood of a monotone missing pattern. There was no significant interaction between treatment condition and baseline externalizing problems. Taken together, the attrition analyses indicated a plausible missing at random mechanism, and missing data could be handled with the full information robust maximum likelihood procedure.

### Implementation

After working 3 years with the N-PALS model, the intervention components and strategies were satisfactorily implemented in most schools (86 to 96%). However, only 1/3 of the schools had reached the 80% threshold for the EBS-SAS subscale targeting individual strategies and supports (Sørlie and Ogden 2015).

### Determining the Number of Trajectory Classes

Fit indices for the one-class solution in the comparison group was RMSEA = .046; 90% CI (.024, .070); CFI = .98; TLI = .98; SRMR = .033. For unconditional (Table 1) and conditional LCGAs, AIC (Akaike information criterion), BIC (Bayesian information criterion), SSBIC (sample size-adjusted Bayesian information criterion), and BLRT (parametric bootstrapped likelihood ratio test) showed better fit for each additional class from two to five classes. The LRT (Lo-Mendel-Rubin likelihood ratio test) favored three to four classes. Unconditional and conditional GMMs supported the same as the LCGAs, except that LRT favored three classes for the unconditional and four classes for the conditional GMM. As the superiority of more classes could simply be a result of their ability to better represent the non-normality of the multivariate distribution of the repeated measures, we wanted to be careful not to overextract classes. Comparing the functional forms of the different class solutions to parsimony, theoretical justification, and interpretability (Jung and Wickrama 2008) made us stop with four, i.e., a stable low trajectory (84.7%, *n* = 1007), a medium but decreasing trajectory (7.4%, *n* = 88), a medium but increasing trajectory (5.5%, *n* = 65), and a high-stable trajectory (2.4%, *n* = 29). This solution corresponded quite well with the study expectations and previous findings (e.g., Brame et al. 2001; Nagin and Odgers 2010; Odgers et al. 2008).

**Table 1:** Class enumeration. Fit indices and entropy for one-to-five-class latent class growth analyses for externalizing behavior—unconditional analyses (without covariates)

Fit indices	1 class	2 classes	3 classes	4 classes	5 classes
Comparison sample					
∆AIC	4516.150	1977.530	720.960	344.390	0.000
∆BIC	4455.180	1931.800	690.470	329.140	0.000
∆SSBIC	4493.300	1960.390	709.540	338.670	0.000
Entropy	–	0.981	0.974	0.974	0.967
LRT *p* value	–	0.352	0.112	0.138	0.486
BLRT *p* value	–	0.000	0.000	0.000	0.000
Intervention sample					
∆AIC	7251.110	2709.780	1025.460	419.640	0.000
∆BIC	7184.720	2659.990	992.260	403.040	0.000
∆SSBIC	7222.850	2688.580	1011.330	412.580	0.000
Entropy	–	0.978	0.968	0.963	0.965
LRT *p* value	–	0.012	0.013	0.154	0.375
BLRT *p* value	–	0.000	0.000	0.000	0.000
Pooled sample					
∆AIC	11,572.446	4503.294	1628.495	661.318	0.000
∆BIC	11,500.147	4449.070	1592.345	643.243	0.000
∆SSBIC	11,538.276	4477.668	1611.410	652.776	0.000
Entropy	–	0.978	0.970	0.961	0.959
LRT *p* value	–	0.022	0.017	0.012	0.368
BLRT *p* value	–	0.000	0.000	0.000	0.000

*AIC* Akaike information criterion; *BIC* Bayesian information criterion; *SSBIC* sample size-adjusted Bayesian information criterion; *LRT* Lo-Mendell-Rubin test; *BLRT* bootstrap likelihood ratio test

Even though some fit indices suggested moving on to a five-group solution, we were reluctant to do this because of the interpretability and parsimony. In a five-group solution, the substantive difference from the four-group solution was that the medium but decreasing group, and as noted by Eisner and Malti (2015), was split into two groups with intercepts very close to each other, and parallel slopes. In comparison to the four-class solution, the five-class solution added little information. Accordingly, the four-group model was selected as the best solution for the comparison group.

For the intervention group, the one-class model fitted the data, RMSEA = .039; 90% CI (.022, .059); CFI = .98; TLI = .98; SRMR = .023. For the unconditional LCGA, the same patterns were found for the relative fit indices as for the comparison group (Table 1). Furthermore, the conditional LCGAs (adding covariates) as well as unconditional and conditional GMMs confirmed these patterns of fit indices and supported the four-group solution for the intervention sample, i.e., a stable low trajectory (84%, *n* = 1568), a medium but decreasing trajectory (10%, *n* = 186), a medium but increasing trajectory (3.5%, *n* = 64), and a high-stable trajectory (2.7%, *n* = 49).

Finally, the joint analysis of the comparison and intervention group supported a four-group solution (Table 1). The joint GMM gave a four-class solution where 84.4% (*n* = 2578) of the sample had a *persistent low* trajectory with an average TRF baseline score of 1.95. This indicates that a consistently low level of externalizing problem behavior was a characteristic of most students. Furthermore, 7.9% (*n* = 241) had a medium but significantly *decreasing* trajectory with an average baseline score of 19.73. These were students who started out with relatively moderate levels of externalizing behavior (11.2 points above the norm of 24.5 for Norwegian 6–13-year-old students; Larsson and Drugli 2011) but who showed declining levels over time. The *increasing* trajectory included 5.3% (*n* = 162) of the students. This subgroup had a relatively low initial mean TRF score of 10.35, but their behavior changed significantly for the worse over time. The fourth subgroup of 2.5% (*n* = 75) had a high-stable trajectory (*persistent high*), starting from the average baseline score of 39.90. Their mean TRF baseline score was in fact 2.16 above the joint 98 percentile Norwegian cutoff for boys and girls in grades 4–7 (Drugli 2009).

Covariate predictions of trajectory class membership by multinomial logistic regression with the persistent low class as a referent revealed that treatment condition was not related to the chance of ending up in a certain class; that is, the log-odds of being in one class versus a reference class did not change by treatment condition (Table A1, Appendix). The same was found for age. However, the significant negative effect of gender found for all three classes (*p* < .001) indicated that boys were more likely to end up in one of the three externalizing classes compared to the stable low class. No significant differences in gender distribution within subgroups were found across treatment condition (Table A2/b, Appendix). The growth factors for the final solution indicated an average baseline TRF score (intercept) and average change (slope) within each subgroup (Table A3, Appendix).

### Testing Intervention Effects

**Fig. 2:**
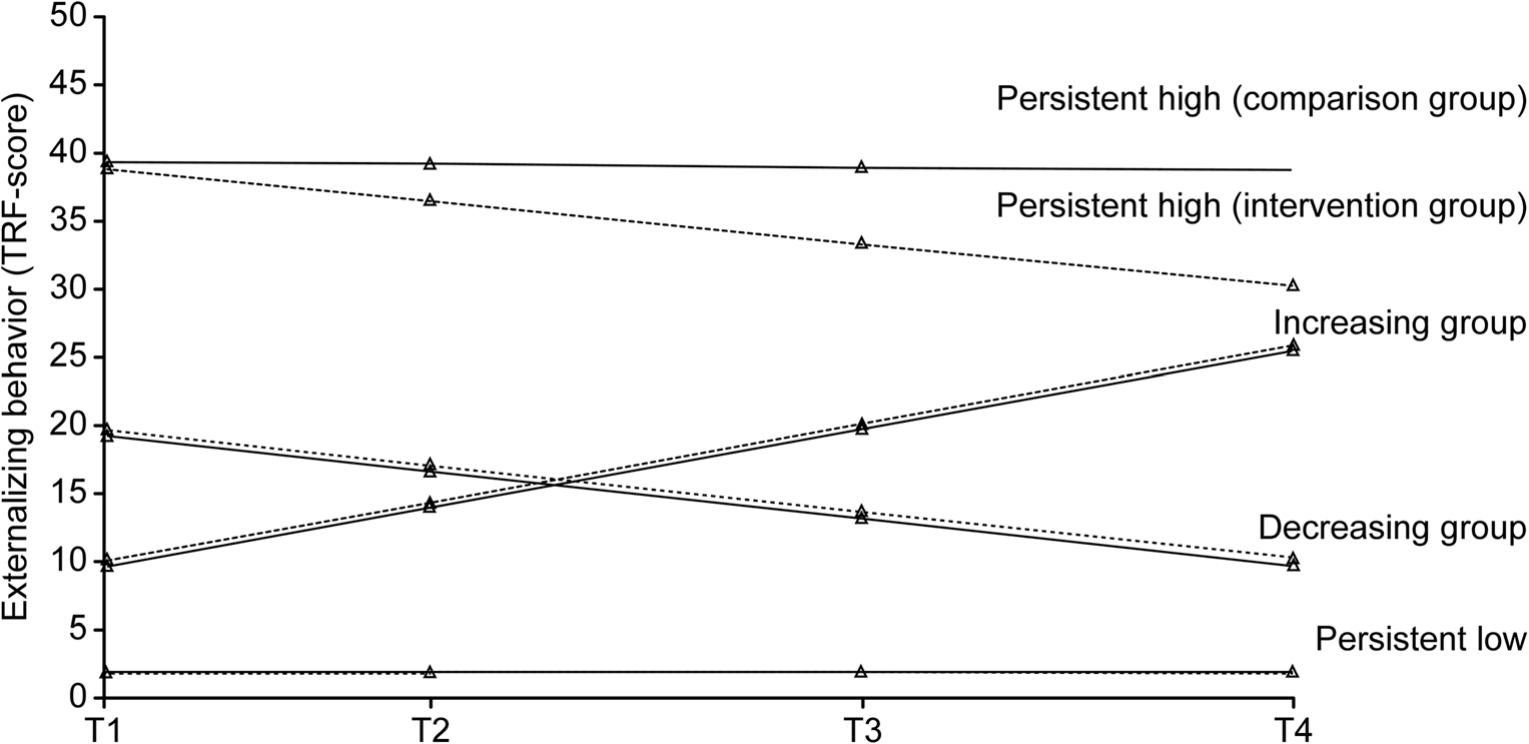
Trajectories of externalizing behavior from grades 4 through 7 for the comparison group (*n* = 1189) and the intervention group (*n* = 1867) with intervention effect for the subgroup persistent high

As can be seen from Table 2, a significant intervention effect of treatment condition on the slopes within the subgroups was found for the *persistent high* class only. The slope for this subgroup was significantly decreasing in the intervention group (*p* = .001), compared to a non-significant change in the control group (*p* = .896, not in table). This is portrayed in Fig. 2 by a decreasing dotted trajectory for the intervention group, while the straight trajectory line for the comparison group was quite stable. A real possibility was that N-PALS produced a change in within-class trajectory different from that expected for controls for the *persistent high* class (Muthén et al. 2002). While the average baseline TRF scores for this class were identical (39.90) within the comparison and intervention group (Table A3, Appendix), the two conditions ended up with significantly different scores (comparison = 38.78, intervention = 30.26) at the end of year 3 after the intervention was initiated. The effect of N-PALS related to this mean difference was medium to strong according to Cohen’s criteria (*d* = − .79, 95% CI = − 1.27, − .31) (Cohen 1988, comparable to differences in persistently high subgroups only).

**Table 2:** Covariate prediction of within trajectory class slope factor

	Persistent											
	Persistent low			Persistent high			Decreasing			Increasing		
Variable	Est.	std	*p*	Est.	std	*p*	Est.	std	*p*	Est.	std	*p*
Gender^a^	0.00	0.00	0.982	2.34	2.19	0.000	− .23	− .83	0.203	0.23	0.82	0.239
Age T1^b^	0.00	0.00	0.974	1.13	1.06	0.004	− .03	− .12	0.879	− .22	− .76	0.506
Treatment condition^c^	0.01	0.03	0.725	− .67	− .63	0.042	0.23	0.86	0.062	0.12	0.43	0.642

*Est.* estimate, *std* standardized solution (*y*); *T1* baseline, ^a^0 = boys; 1 = girls, ^b^0 = grade 4, 1 = grade 5, ^c^0 = comparison group, 1 = intervention group

## Discussion

### Behavioral Trajectories

The current study made us conclude with four distinct teacher-rated latent trajectories of externalizing behavior over four time points from 4th through 7th grade, called *persistent low* (84.4%), *decreasing* (7.9%), *increasing* (5.3%), and *persistent high* (2.5%). Participants were students from 28 schools implementing the school-wide N-PALS (SWPBS) model and students from 20 comparison schools with regular practice. Prediction analyses indicated that boys were more likely than girls to end up in the three most externalizing trajectory groups. Children in high-stable and increasing trajectory groups (here 7.9%) are likely to be at an elevated risk of school failure, escalating social problems, and persistent conduct problems in adolescence and young adulthood (e.g., Bongers et al. 2008; Reef et al. 2011).

The identification of four subgroups of children with significantly different behavioral trajectories corresponds well with findings reported in prior experimental (e.g., Bradshaw et al. 2015) and non-experimental longitudinal research (e.g., Jennings and Reingle 2012; Piquero et al. 2012; Reef et al. 2011). The shape of the behavioral trajectories and the overrepresentation of boys in trajectory classes with increasing externalizing behaviors also correspond well with prior research (e.g., Bradshaw et al. 2015; Farell et al. 2013; Jennings and Reingle 2012; Piquero et al. 2012). Similar results are reported although different statistical methods have been applied, indicating robustness of the present study findings. To our understanding, using several assessment points to identify subgroups with differing behavioral trajectories provides a more valid basis for prevention and intervention efforts than identification based on baseline assessment only because the behavioral instability during preadolescence is better taken into account.

### Differential Effects of N-PALS

Although the baseline scores of the high-risk students (*persistent high*) were identical in the intervention and comparison groups, the trajectory class in the N-PALS schools showed a significantly larger drop in problem behavior over time than their counterparts in the comparison schools. The finding indicates that the N-PALS interventions were more effective in reducing externalizing problem behaviors in this trajectory class than those applied in the comparison schools. The effect was in the moderate-to-large range, which indicates a benefit of practical significance. The fact that the students with the most troublesome behavioral trajectory changed the most following the N-PALS model is also supported by findings in prior studies of school-based interventions (e.g., Farrell et al. 2001; Simon et al. 2008).

The *increasing* trajectory demonstrated that some students behaved worse over time, according to teacher ratings. Contrary to Bradshaw et al. (2015) study, it did not seem that these students responded as expected to the interventions, maybe because these were not effectively implemented or sufficiently adequate. Students with escalating externalizing problem behavior may have been susceptible to negative peer influences, exposed to stress and conflicts at home, initially overlooked for advanced supports, or maybe they responded negatively to the model’s structured approach. We cannot rule out the possibility that some students may have experienced the intervention model negatively because it redirected some of the teachersʼ attention to other students, or that they may have felt that their behavioral freedom decreased. A closer examination of this group is warranted in order to find out if their paradoxical reaction was related to any common contextual or individual characteristics which distinguished them from the other students.

No differences in externalizing behavior were found between students in the *decreasing* trajectory over time when the treatment conditions were compared. Their medium but decreasing trajectory indicated that they improved behaviorally over time, regardless of what school group they belonged to. The extra support and structured environment of the N-PALS model did not seem to benefit these students, and they adapted equally well to the expectations of the intervention as in the comparison schools. One might speculate if this group’s positive behavioral change, at least partly, may have been due to individual maturation or advantageous changes in their lives outside the school context (Ben-Arieh et al. 2014; Eisner and Malti 2015).

Finally, and as expected, no significant group differences were found for the majority of students with a *persistent low* trajectory. This group of students gives reason to question whether a whole-school intervention is warranted when most of the students are obviously not at risk. But it may still be important and fair that all students should experience universal interventions such as positive expectations and behavior support, social skills training, and research-based teaching methods. Such interventions may have long-term impacts on the majority of students and prevent student recruitment to more intensive interventions.

### Limitations

Although the present study sheds light on behavioral development during middle childhood and on the effectiveness of the SWPBS model as implemented in Norway, certain limitations should be mentioned. Even if the high-risk group improved more in the N-PALS schools than in the comparison schools, it was not possible to identify which components or parts of the model caused this change. According to implementation quality scores measured after year 3, individually tailored interventions were only to some extent implemented in the N-PALS schools. However, we do not know precisely how many and which supports were applied to which students. This limitation also pertains that no clear cutoff criteria are established to decide which students should be considered eligible for interventions at the selected and indicated level. Moreover, only teacher assessments were used. Teacher ratings have been shown reliable (Ogden 2003), but adding other informants might have increased the validity of the findings. Only one outcome variable was targeted. Although externalizing behavior is the main outcome variable in N-PALS, changes in other student variables might have been relevant (e.g., social competence). As noted by Little et al. (2009), researchers should be cautious when using GMM. They underscore principles of interpretability and replicability and recommend a strong theory to support that the framework is confirmatory. Incidentally, the analyses did not capture the potential for shift of members across groups, which may have masked even larger differences between conditions. As the four-group solution based on theory underlying SWPBS and Moffittʼs theoretical and empirical framework (Moffitt 1993) has been identified in several previous studies (Eisner and Malti 2015), we suggest this as a recommendable and practically relevant solution based on the current data. However, we recognize the exploratory nature of GMM, and readers are encouraged to interpret the findings with care.

## Conclusions and Future Implications

Four different trajectories of externalizing problem behavior were identified in a sample of typically developing children, describing distinct groups of students who have differing needs of behavioral supports in school. The results indicate that those students who were considered to be most at risk for later conduct disorder, school failure, and other negative life outcomes benefitted the most from the SWPBS model as implemented in Norway. The results indicate that students at elevated risk may benefit substantially from universal interventions. However, to contribute more effectively to the prevention of serious behavioral problems in children and youth, schools should preferably combine sustained research-supported universal interventions with targeted interventions matched to the studentsʼ individual needs and risk level (e.g., Weare and Nind 2011). Taken together, the study results are encouraging but warrant replication in future longitudinal effectiveness studies using robust analytical approaches before more firm conclusions of heterogeneous behavior trajectories during middle childhood and student outcomes of the SWPBS/N-PALS model can be drawn.

## Electronic supplementary material

ESM 1 (DOCX 130 kb)

ESM 2 (PNG 76 kb)

High Resolution Image (TIF 397 kb)

## References

[CR1] Achenbach TM (1991). Manual for Teacher’s Report Form and 1991 Profile.

[CR2] Augimeri LK, Farrington DP, Koegl CJ, Day DM (2007). The SNAP™ under 12 outreach project: Effects of a community-based program for children with conduct problems. Journal of Child and Family Studies.

[CR3] Baker SG, Kramer BS (2001). Good for women, good for men, bad for people. Simpsonsʼs paradox and the importance of sex-specific analysis in observational studies. Journal of Womenʼs Health & Gender-Based Medicine.

[CR4] Ben-Arieh A, Casa F, Froenes I, Korbin JE (2014). Handbook of child well-being. Theories, methods and policies in global perspective.

[CR5] Bongers IL, Koot HM, van der Ende J, Verhulst FC (2008). Predicting young adult social functioning from developmental trajectories of externalizing behavior. Psychological Medicine.

[CR6] Bradshaw CP, Reinke WM, Brown LD, Bevans KB, Leaf PJ (2008). Implementation of school-wide positive behavior interventions and supports (PBIS) in elementary schools: Observations from a randomized trial. Education and Treatment of Children.

[CR7] Bradshaw CP, Wassdorp TE, Leaf PJ (2015). Examining variation in the impact of school-wide positive behavioral interventions and supports: Findings from a randomized controlled effectiveness trial. Journal of Educational Psychology.

[CR8] Brame B, Nagin DS, Tremblay RE (2001). Developmental trajectories of physical aggression from school entry to late adolescence. Journal of Child Psychology and Psychiatry.

[CR9] Broidy LM, Nagin DS, Tremblay RE, Bates JE, Brame B, Dodge KA (2003). Developmental trajectories of childhood disruptive behaviors and adolescent delinquency: A six-site, cross-national study. Developmental Psychology.

[CR10] Cohen J (1988). Statistical power analysis for the behavioral sciences.

[CR11] Drugli, M. B. (2009). Norwegian cutoffs for high-risk boys and girls in grades 1 to 3 and grades 4 to 7 on the externalizing scale of the Teacher Report Form. Personal communication, e-mail: September 30, 2009.

[CR12] Eisner, M. P., & Malti T. (2015). Aggressive and violent behavior. In M. E. Lamb (Vol Ed.) and R. M. Lerner (Series Ed.), *Handbook of child psychology and developmental science, Vol. 3: Social, emotional and personality development* (7th ed., pp. 795–884). New York: Wiley.

[CR13] Enders CK (2010). Applied missing data analysis.

[CR14] Farell AD, Henry DB, Bettencourt A (2013). Methodological challenges examining subgroup differences: Examples from universal school-based youth violence prevention trials. Prevention Science.

[CR15] Farrell AD, Meyer AL, White KS (2001). Evaluation of responding in peaceful and positive ways (RIPP): A school-based prevention program for reducing violence among urban adolescents. Journal of Clinical Child Psychology.

[CR16] Flay Brian R., Biglan Anthony, Boruch Robert F., Castro Felipe González, Gottfredson Denise, Kellam Sheppard, Mościcki Eve K., Schinke Steven, Valentine Jeffrey C., Ji Peter (2005). Standards of Evidence: Criteria for Efficacy, Effectiveness and Dissemination. Prevention Science.

[CR17] Harachi TW, Fleming CB, White HR, Ensminger ME, Abbott RD, Catalano RF, Haggerty KP (2006). Aggressive behavior among girls and boys during middle childhood: Predictors and sequelae of trajectory group membership. Aggressive Behavior.

[CR18] Hu J, Leite WL, Gao M (2017). An evaluation of the use of covariates to assist in class enumeration in linear growth mixture modeling. Behavior Research Methods.

[CR19] Humphrey N, Lendrum A, Wigelsworth M (2013). Making the most out of school-based prevention: Lessons from the social and emotional aspects of learning (SEAL) programme. Emotional and Behavioural Difficulties.

[CR20] Husemann LR, Dubow EF, Boxer P (2009). Continuity of aggression from childhood to early adulthood as a predictor of life outcomes. Implications for the adolescent-limited and life-course-persistent models. Aggressive Behavior.

[CR21] Jennings WG, Reingle JM (2012). On the number and shape of developmental/life-course violence, aggression, and delinquency trajectories. A state-of-the-art review. Journal of Criminal Justice.

[CR22] Jung T, Wickrama KAS (2008). An introduction to latent class growth analysis and growth mixture modeling. Social and Personality Psychology Compass.

[CR23] Larsson M, Drugli MB (2011). School competence and emotional/behavioral problems among Norwegian school children as rated by teachers on the Teacher Report Form. Scandinavian Journal of Psychology.

[CR24] Little TD, Card NA, Preacher KJ, McConnell E, Lerner RM, Steinberg L (2009). Modeling longitudinal data from research on adolescence. Handbook of adolescent psychology.

[CR25] Moffitt TE (1993). Adolescent-limited and life-course-persistent antisocial behavior: A developmental taxonomy. Psychological Review.

[CR26] Muthén, B., & Muthén, L. (2014). *Mplus 7.2*. Los Angeles. www.statmodel.com.

[CR27] Muthen B. (2002). General growth mixture modeling for randomized preventive interventions. Biostatistics.

[CR28] Nagin D, Odgers CL (2010). Group-based trajectory modeling in clinical research. Annual Review of Clinical Psychology.

[CR29] Odgers CL, Moffitt TE, Broadbent JM, Dickson N, Hancox RJ, Harrington H (2008). Female and male antisocial trajectories: From childhood origins to adult outcomes. Development and Psychopathology.

[CR30] Ogden T (2003). The validity of teacher ratings of adolescents’ social skills. Scandinavian Journal of Educational Research.

[CR31] Ogden T (2015). Sosial komeptanse og problematferd blant barn og unge.

[CR32] Ogden T, Hagen KA (2006). Multisystemic treatment of serious behavior problems in youth: Sustainability of effectiveness two years after intake. Child and Adolescent Mental Health.

[CR33] Ogden T, Hagen KA (2014). Adolescent mental health. Prevention and intervention.

[CR34] Piquero AR, Carriga ML, Diamond B, Kazemian L, Farringgton DP (2012). Stability in aggression revisited. Aggression and Violent Behavior.

[CR35] Reef J, Diamantopoulou S, van Meurs I, Verhulst FC, van der Ende J (2011). Developmental trajectories of child to adolescent externalizing behavior and adult DSM-IV disorder: Results of a 24-year longitudinal study. Social Psychiatry and Psychiatric Epidemiology.

[CR36] Schulz KF, Altman DG, Moher D (2010). Consort 2010 statement: Updated guidelines for reporting parallel group randomized trials. BMJ.

[CR37] Shadish WR, Cook TD, Campbell DT (2002). Experimental and quasi-experimental designs for generalized causal inference.

[CR38] Simon TR, Ikeda RM, Smith EP, Reese LRE, Rabiner DL, Miller-Johnson S (2008). The multisite violence prevention project: Impact of a universal school-violence prevention program on social-cognitive outcomes. Prevention Science.

[CR39] Solomon BG, Tobin KG, Schutte GM (2015). Examining the reliability and validity of the Effective Behavior Support Self-Assessment Survey. Education and Treatment of Children.

[CR40] Sørlie M-A, Ogden T (2014). Reducing threats to validity by design in a nonrandomized experiment of a school-wide prevention model. International Journal of School & Educational Psychology.

[CR41] Sørlie, M-A., & Ogden, T. (2015). School-Wide Positive Behavior Support - Norway: Impacts on problem behavior and classroom climate*. International Journal of School & Educational Psychology, 00*, 1–16.

[CR42] Sørlie, M-A., & Ogden, T., & Olseth, A. R. (2016). Examining teacher outcomes of the School-Wide Positive Behavior Support model in Norway*. SAGE Open, 00*, 1–13. 2158244016651914.

[CR43] Split JL, Koot JM, van Lier PAC (2013). For whom does it work? Subgroup differences in the effects of a school-based universal prevention program. Prevention Science.

[CR44] Sugai, G., Horner, R. H., & Todd, A. W. (2009). Effective behavior support (EBS) self-assessment survey (version 3). Eugene: University of Oregon, Educational and Community Supports. https://www.pbisapps.org/Applications/Pages/PBIS-Assessment-Surveys.aspx#sas.

[CR45] Todd AW, Campbell AL, Meyer GG, Horner RH (2008). The effects of a targeted intervention to reduce problem behaviors: Elementary school implementation of check in—check out. Journal of Positive Behavior Interventions.

[CR46] Tremblay RE (2000). Origins of youth violence. Canadian Journal of Policy Research.

[CR47] Walters GD, Ruscio J (2013). Trajectories of youthful antisocial behavior: Categories or continua?. Journal of Abnormal Child Psychology.

[CR48] Weare K, Nind M (2011). Mental health promotion and problem prevention in schools: What does the evidence say?. Health Promotion International.

