# Supplementary material for: Behavioral Trajectories During Middle Childhood: Differential Effects of the School-Wide Positive Behavior Support Model
Source: Prev Sci. 2018 Aug 18;19(8):1055–65. doi: 10.1007/s11121-018-0938-x (PMC6208577; doi:10.1007/s11121-018-0938-x)
Supplement: Supplementary file 1 — (DOCX 130 kb) [file 11121_2018_938_MOESM1_ESM.docx]

**APPENDIX**

to

“Behavioral Trajectories during Middle Childhood: Differential Effects of the School-Wide Positive Behavior Support Model”


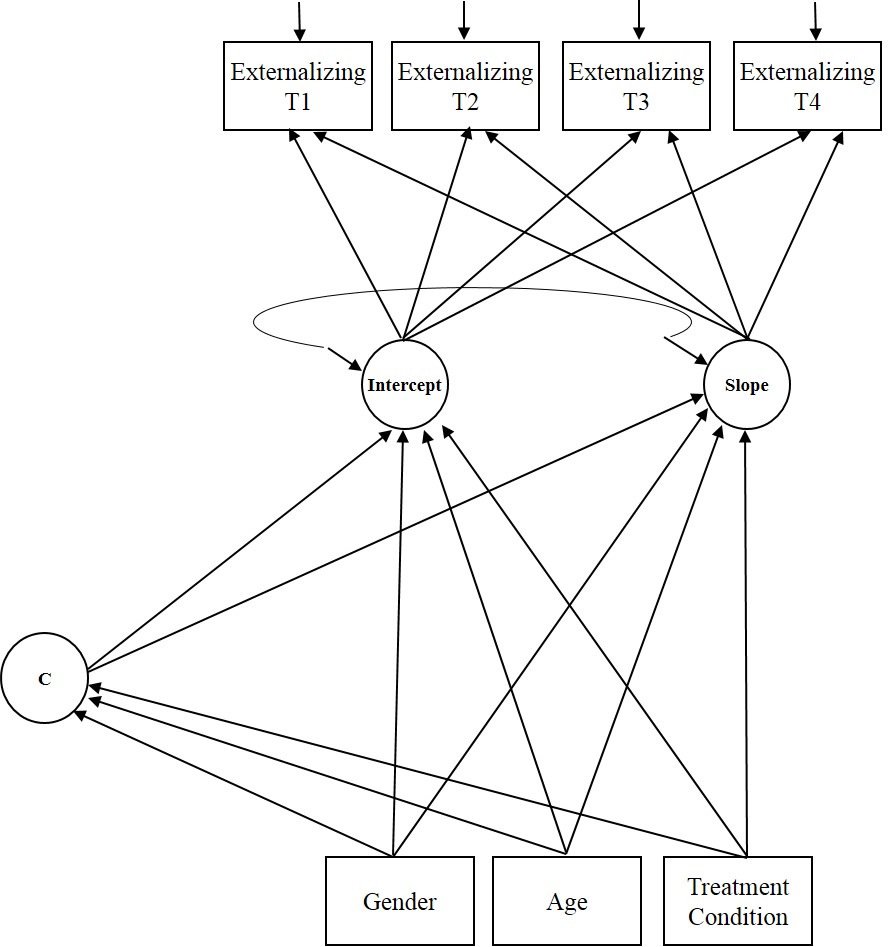


Figure A1

*Conceptual model*

The figure describes the conceptual model of a growth mixture model (GMM) for Externalizing Behavior measured four times from grades four through seven. C is a categorical variable representing subgoups (classes), and the arrows from c to the intercept (baseline) and slope (change) indicate that the intercepts in the regressions of those growth factors on the three X variables (gender, age and treatment condition) may vary across the classes of c. The arrows from the three independent variables to c represent multinomial logistic regressions of c on the independent variables. This model enables us to investigate potential differential distributions within classes when it comes to gender, age and treatment condition. Furthermore, the model allows us to inspect potential baseline differences (arrows from the independent variables to the intercept factor) and potential intervention effects (arrow from Treatment Condition to the slope factor) after controlling for gender and age.

Table A1

*Covariate Prediction of Trajectory Class Membership. Multinomial Logistic Regression*

| *Persistent High Decreasing Increasing* | | | | | | | | | | | |
| --- | --- | --- | --- | --- | --- | --- | --- | --- | --- | --- | --- |
| Variable | *OR* | *SE* | *p* |  | *OR* | *SE* | *p* |  | *OR* | *SE* | *p* |
| Gender _a_ | -1.99 | .32 | .000 |  | -1.26 | .21 | .000 |  | -.95 | .26 | .000 |
| Age T1 _b_ | -.62 | .31 | .047 |  | -.38 | .23 | .093 |  | -.18 | .34 | .594 |
| Treatment Condition _c_ | -.01 | .32 | .981 |  | .17 | .18 | .360 |  | -.12 | .27 | .669 |

*Note*. The *Persistent Low* class served as the referent. OR = odds ratio, SE = standard error; T1 = baseline.

a 0 = boys; 1 = girls; b 0 = grade 4, 1 = grade 5; c 0 = comparison group, 1 = intervention group

Table A2

*Gender Distribution for 4-Class Growth Mixture Model*

|  |  |  | *Gender* | | | |
| --- | --- | --- | --- | --- | --- | --- |
| Class | Treatment Condition |  | Girls | | Boys | |
|  |  |  | *%* | *n* | *%* | *n* |
| Persistent Low (84.4%) | Intervention group |  | 55.7 | 876 | 44.3 | 698 |
|  | Comparison group |  | 53.2 | 536 | 46.8 | 472 |
| Persistent High (2.5%) | Intervention group |  | 15.2 | 7 | 84.8 | 39 |
|  | Comparison group |  | 13.3 | 4 | 86.7 | 26 |
| Decreasing (7.9%) | Intervention group |  | 23.2 | 36 | 76.8 | 119 |
|  | Comparison group |  | 29.1 | 25 | 70.9 | 61 |
| Increasing (5.3%) | Intervention group |  | 29.3 | 27 | 70.7 | 65 |
|  | Comparison group |  | 30.8 | 20 | 69.2 | 45 |

Table A2b

*Observed Baseline scores for Externalizing Behavior for 4-Class Growth Mixture Model by Treatment Condition and Gender ^a^*

|  | | *Average score* | | |
| --- | --- | --- | --- | --- |
| Class | Treatment Condition | Pooled | Girls | Boys |
| Persistent Low (84.4%) | Intervention group | 1.89 | 1.23 | 2.72 |
|  | Comparison group | 1.82 | 1.27 | 2.46 |
| Persistent High (2.5%) | Intervention group | 38.98 | 35.2 | 39.46 |
|  | Comparison group | 38.43 | 23.67^b^ | 40.20 |
| Decreasing (7.9%) | Intervention group | 18.77 | 16.46 | 19.52 |
|  | Comparison group | 20.25 | 22.00 | 19.51 |
| Increasing (5.3%) | Intervention group | 10.42 | 5.04 | 12.64 |
|  | Comparison group | 10.61 | 5.35 | 12.85 |

*Note*. ^a^ Based on scores obtained from analyses with valid n after list-wise deletion. Class enumeration procedures are conducted under the MAR assumption with FIML estimation in Mplus.

^b^ Only four girls in this subgroup, and among them outliers down to scores of 17 (note that tests for outliers did not affect the class enumeration procedures as reported in the article).

Table A3

*Growth Factor Parameter Estimates for 4-Class Growth Mixture Model*

| *Intercept Slope* | | | | | | | |
| --- | --- | --- | --- | --- | --- | --- | --- |
| Class | *Est.* | *SE* | *p* |  | *Est.* | *SE* | *p* |
| Persistent Low (84.4%, n = 2,582) | 1.95 | .17 | .000 |  | .01 | .02 | .682 |
| Persistent High (2.5%, n = 76) | 39.90 | 2.47 | .000 |  | -.58 | .42 | .167 |
| Decreasing (7.9%, n = 241) | 19.73 | 2.32 | .000 |  | -.87 | .16 | .000 |
| Increasing (5.3%, n = 157) | 10.35 | 1.22 | .000 |  | 1.50 | .18 | .000 |

*Note.* Est. = estimate, SE = standard error
