# Supplementary figures and images for: Behavioral Trajectories During Middle Childhood: Differential Effects of the School-Wide Positive Behavior Support Model
Source: Prev Sci. 2018 Aug 18;19(8):1055–65. doi: 10.1007/s11121-018-0938-x (PMC6208577; doi:10.1007/s11121-018-0938-x)

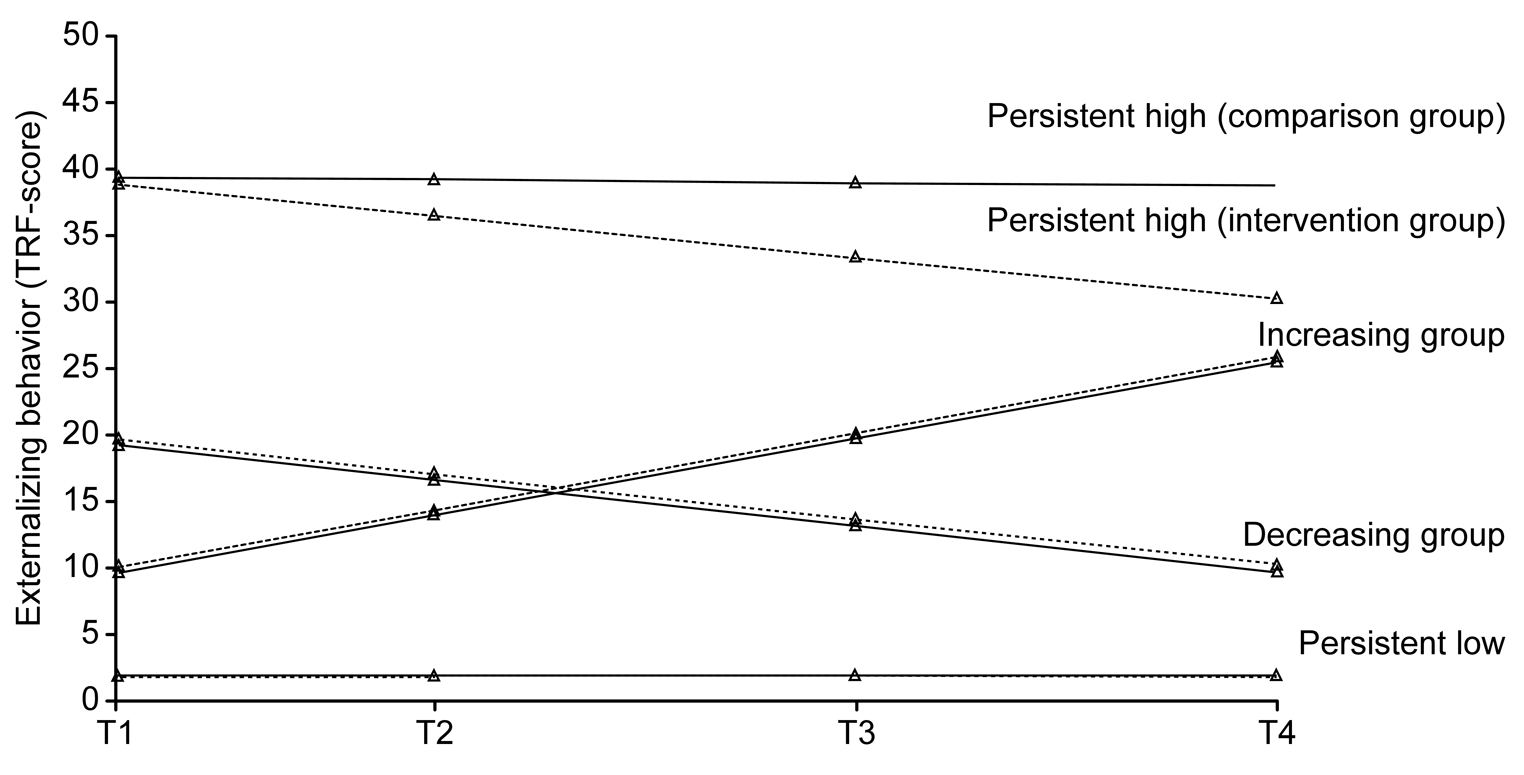

Supplement: Supplementary file 2 — (PNG 76 kb) [file 11121_2018_938_Fig3_ESM.png]
